# Editorial: Insights in surgical ophthalmology: 2023

**DOI:** 10.3389/fopht.2024.1407362

**Published:** 2024-04-17

**Authors:** Yongwei Guo, Ludwig M. Heindl

**Affiliations:** ^1^ Eye Center, The Second Affiliated Hospital, School of Medicine, Zhejiang University, Zhejiang Provincial Key Laboratory of Ophthalmology, Zhejiang Provincial Clinical Research Center for Eye Diseases, Zhejiang Provincial Engineering Institute on Eye Diseases, Hangzhou, Zhejiang, China; ^2^ Department of Ophthalmology, Faculty of Medicine and University Hospital of Cologne, University of Cologne, Cologne, Germany; ^3^ Center for Integrated Oncology (CIO) Aachen-Bonn-Cologne-Duesseldorf, Cologne, Germany

**Keywords:** ocular surface, keratoplasty, ectropion, refractive surgery, glaucoma, cataract, posterior eye segment, strabismus

The discipline of Surgical Ophthalmology, as a distinct branch within ophthalmology, aims to alleviate patients’ complaints through a range of surgical procedures. Recently, remarkable strides have been made in the rapidly evolving domain of surgical ophthalmology. Our Research Topic, titled “*Insights in surgical ophthalmology: 2023*”, explores the cutting-edge research and advancements in this field, emphasizing novel perspectives, contemporary obstacles, future prospects, and sophisticated surgical techniques that promise to contribute to the ongoing progress within Surgical Ophthalmology. Consequently, Guo et al. emphasized the most pivotal challenges and recent advancements across all ophthalmic subspecialties, inspiring, informing, guiding, and motivating ophthalmologists to enhance their medical practices to improve patient care.

Ocular surface disorders (OSD), which are highly prevalent, can significantly impact vision quality and overall quality of life, potentially leading to blindness in extreme cases. However, some treatments may be ineffective due to a limited understanding of OSD mechanisms. Fortunately, surgical advancements such as simple limbal epithelial transplantation (SLET) (1), corneal endothelial transplantation (2), and corneal neurotization for neurotrophic keratitis (3), among others, have significantly improved the management of severe OSDs. Innovative imaging technologies are also emerging as potential tools to enhance OSD prognosis. During Descemet membrane endothelial keratoplasty (DMEK) surgery, intraoperative optical coherence tomography (iOCT) provides crucial feedback for graft positioning. A recent study by Muijzer et al. involved a prospective, non-inferiority, international multicenter randomized controlled trial with 65 pseudophakic eyes of patients with Fuchs endothelial dystrophy scheduled for routine DMEK. The study found that iOCT-guided DMEK surgery, which avoids prolonged over-pressurization, was at least as effective as the traditional method. Although the overall rate of adverse postoperative events remained unchanged, the surgery and unfolding times decreased. They presented iOCT’s beneficial role in 40% of cases and considered it indispensable in 9% of the traditional cases.

Recently, ophthalmic plastic and reconstructive surgery has witnessed rapid progress, encompassing various advancements. These include utilizing biopharmaceuticals for inflammatory conditions of the orbit and recurrent cancers, employing Artificial Intelligence (AI) in planning perioperative treatments and evaluating surgical outcomes (4–7)), and developing minimally invasive surgical techniques. Among these techniques, endoscopic orbital surgery combined with 3D stereo navigation and laser-assisted procedures for lacrimal diseases (8–10), stand out. In another noteworthy study of this Research Topic, Kopecký et al. examined the advancements in the lateral tarsal strip, a technique widely used, especially in the repair of ectropion. They presented its alternative approaches and discussed the future prospects of this technique, providing valuable insights and guidance to researchers in this field.

Modern refractive surgical techniques have undergone significant advancements in terms of both safety and effectiveness in achieving desired visual and refractive outcomes. Chen et al. conducted a study to assess the influence of short white-to-white corneal diameters (10.6 mm) on the safety and anterior chamber structure changes in patients who underwent the Evolution implantable Collamer lens implantation. The results revealed that the patients achieved an optimal vault, normal-range changes in the anterior chamber structure, and stable intraocular pressure (IOP) following the surgery. Based on these findings, the authors concluded that the procedure is safe, efficient, and reliable for correcting myopia in this patient population.

Glaucoma represents a collection of degenerative optic nerve conditions that ultimately result in irreversible blindness. The condition is characterized by the loss of retinal ganglion cells, the thinning of the retinal nerve fiber layer, and the formation of a cupped optic disc. Recent advancements in targeted therapeutics, such as the prostaglandin-EP2 agonist (omidenepag isopropyl OMDI), have exhibited encouraging clinical responses with minimized adverse effects. Additionally, minimally invasive glaucoma surgeries (MIGSs) have emerged as promising alternatives, offering reduced risks of complications and similar intraocular pressure (IOP) reduction. In a meta-analysis conducted by Raja et al., the safety and effectiveness of the Molteno glaucoma implant were compared to the Ahmed glaucoma valve across four studies involving 257 patients with refractory, neovascular, or advanced uncontrolled glaucoma. Their findings revealed non-significant differences in the primary outcome between patients receiving the Molteno glaucoma implant and Ahmed glaucoma valve. However, regarding secondary outcomes, the Ahmed glaucoma valve demonstrated a decreased need for anti-glaucoma medication and a significant reduction in complications.

Moreover, cataract surgery is performed on approximately 20 million individuals globally annually, ranking it as one of the most frequently conducted surgeries worldwide (11). Numerous innovative surgical techniques have significantly enhanced the treatment of cataracts, including femtosecond laser-assisted cataract surgery (FLACS) and advanced intraocular lens (IOL) options such as multifocal and accommodative IOLs. The posterior segment of the eye, encompassing the rear two-thirds of the ocular structure, has also undergone remarkable improvements. Rapid advancements in fundus imaging technology, the availability of anti-VEGF medications, and innovative minimally invasive vitrectomy procedures have collectively led to significant progress in managing common retinal diseases. Concerning strabismus, surgical success hinges on meticulous surgical planning. As a result, recent years have witnessed numerous innovations and modifications in surgical strategies for treating common strabismus (e.g., basic-type intermittent exotropia) and more complex cases like paralytic strabismus (e.g., abducens nerve palsy).

Surgical Ophthalmology encompasses a wide range of ocular structures and associated tissues, offering a comprehensive understanding of the surgical practices involved. This Research Topic presents invaluable perspectives that can facilitate the ongoing enhancement of surgical ophthalmology, highlighting the most recent advancements and fostering further exploration and progress in this domain. We anticipate that it will significantly enhance patient care and surgical outcomes across all subdomains of Surgical Ophthalmology, providing a captivating reading experience for our esteemed readers.

## Author contributions

YG: Conceptualization, Data curation, Formal analysis, Investigation, Methodology, Project administration, Software, Validation, Visualization, Writing – original draft. LH: Conceptualization, Methodology, Resources, Supervision, Validation, Writing – review & editing.
